# An Intelligent Knowledge-Based and Customizable Home Care System Framework with Ubiquitous Patient Monitoring and Alerting Techniques

**DOI:** 10.3390/s120811154

**Published:** 2012-08-10

**Authors:** Yen-Lin Chen, Hsin-Han Chiang, Chao-Wei Yu, Chuan-Yen Chiang, Chuan-Ming Liu, Jenq-Haur Wang

**Affiliations:** 1 Department of Computer Science and Information Engineering, National Taipei University of Technology, 1, Sec. 3, Chung-hsiao E. Rd., Taipei 10608, Taiwan; E-Mails: ylchen@csie.ntut.edu.tw (Y.-L.C.); david741002@gmail.com (C.-W.Y.); cmliu@csie.ntut.edu.tw (C.-M.L.); 2 Department of Electrical Engineering, Fu Jen Catholic University, New Taipei City 24205, Taiwan; E-Mail: hsinhan@ee.fju.edu.tw; 3 Department of Computer Science, National Chiao Tung University, 1001 University Road, Hsinchu 30050, Taiwan; E-Mail: gmuooo@gmail.com

**Keywords:** Healthcare systems, knowledge-based systems, patient monitoring, handheld devices, component-based framework, ubiquitous monitoring

## Abstract

This study develops and integrates an efficient knowledge-based system and a component-based framework to design an intelligent and flexible home health care system. The proposed knowledge-based system integrates an efficient rule-based reasoning model and flexible knowledge rules for determining efficiently and rapidly the necessary physiological and medication treatment procedures based on software modules, video camera sensors, communication devices, and physiological sensor information. This knowledge-based system offers high flexibility for improving and extending the system further to meet the monitoring demands of new patient and caregiver health care by updating the knowledge rules in the inference mechanism. All of the proposed functional components in this study are reusable, configurable, and extensible for system developers. Based on the experimental results, the proposed intelligent homecare system demonstrates that it can accomplish the extensible, customizable, and configurable demands of the ubiquitous healthcare systems to meet the different demands of patients and caregivers under various rehabilitation and nursing conditions.

## 1. Introduction

Healthcare approaches and systems for older and chronic disease patients are a vital research field. Because of heavy workloads for careered people in industrialized nations, maintaining long-term and personal homecare for their older or chronically sick family members has become a difficult challenge. With the technical development of information and communications technology (ICT), new homecare techniques and products based on ICT, such as wireless emergency telemedicine systems, older adults' home emergency rescuing systems, infant-monitoring systems, mobile healthcare systems, and handheld electronic patient records, have progressively entered contemporary family life.

Currently, personal demands for providing healthcare services to patients differ by case according to health, age, and disease conditions in various rehabilitation environments or nursing settings. Thus, flexible intelligent home healthcare systems should integrate multiple physiological sensors for monitoring and caring for patients, as well as provide customized functions for caregivers or system developers to design the most favorable healthcare systems for patients with various healthcare demands. To achieve these purposes, a healthcare system that can efficiently and rapidly monitor the daily physiological and medication treatment procedures of patients based on the information obtained from different multiple physiological sensors (such as body temperature, heart rate, blood pressure, blood oxygen values, breath meters, and ECGs) and camera sensors are required for various healthcare demands. The information from these physiological sensors can then be monitored and used by caregivers or nursing home staff to detect possible accidents and emergencies of patients [1–10].

In recent healthcare and nursing studies, because of the high advancement of network technology, Internet-based homecare systems have been developed to provide long-term health care and monitoring by transmitting physiological data obtained from the biosensors worn by patients or infants through the Internet to caregivers or nursing centers [1–3]. However, these healthcare systems mostly limit the activity regions of patients and their caregivers to residences or healthcare centers. Because of the rapid development of wireless communication technology, wireless communication devices and newly developed healthcare systems can afford more freedom to patients and caregivers. Recently, some healthcare systems have adopted wireless and mobile communication techniques [4–10], such as radio-frequency identification (RFID), wireless communication networks (WIFI), global systems for mobile communications (GSM), and general packet radio service (GPRS), to monitor and record the physiological data acquired by sensors worn by patients. Patient physiological data records are then transmitted to caregivers or healthcare centers via wireless communication networks to remote terminal devices or systems (such as personal digital assistants, PDAs), mobile phones, or healthcare center servers). Recently, the bandwidths of mobile communications have dramatically expanded because of the development of third generation/fourth generation (3G/4G) mobile communication technologies, as well as the advanced computing power of newly released mobile communication devices. Thus, transmitting and playing videos on handheld devices has become feasible for remote monitoring of patient rehabilitation activities.

In emerging research, integration of multiple sensors, as well as the corresponding artificial intelligence techniques for sensor data fusion and processing, ambient event recognition, affective computing, and intelligent automation, within a spread environment for assisting people's daily life, has become an emerging research topic called “ambient intelligence” [11,12]. In recent studies, researchers have applied and developed the ambient intelligence concepts in many applications by adopting multiple sensors, such as human-computer interactions [13,14], pedestrian protection in traffic environments [15], intelligent driver assistance systems [16,17], patient disease diagnosis support systems [18,19], and patient activity recognition in healthcare environments [20,21].

However, most of the mentioned present healthcare techniques and systems are designed for specific health care objectives and function for patients with predetermined diseases or rehabilitation needs; thus, the flexibility and feasibility of these systems are limited for adapting adequately to diverse demands and environments of various healthcare services, caregivers, and patients. Furthermore, these conventional systems have generally provided neither remote video-monitoring functions for the mobile handheld devices of caregivers, nor a customized and configurable framework for intelligent home healthcare and video-monitoring systems. To provide the flexibility and extensibility for various demands on intelligent healthcare systems, knowledge-based system technologies have demonstrated the advantages of extensibility and flexibility for a wide variety of practical applications [22,23]. Consequently, many researchers have recently applied the concepts of knowledge-based systems to many applications, such as scene analysis [24], document analysis for office automation [25,26], automatic traffic monitoring [27], facial analysis for human-computer interaction [28], clinical decision supporting systems [29,30], and healthcare information systems [31,32].

Nazif and Levine [24] proposed an efficient and flexible three-level knowledge-based model for low-level scene analysis applications. In previous studies on document image analysis, Niyogi and Srihari [25] and Chen *et al.* [26] also applied the concepts of knowledge-based systems for efficient text content and geometric analysis of various document images for office automation applications, such as color documents, newspapers, magazines, and compound documents with mixed text/graphics contents. Cucchiara *et al.* [27] applied knowledge-based systems to real-time video-based traffic-monitoring applications. Subasic *et al.* [28] presented an expert system-based facial detection system for human-computer interactions that integrates a low-level image segmentation module and a multistage rule-based labeling system. In Lee *et al.*'s clinical decision support system [29], the knowledge-based processing system was integrated with a workflow management system to provide clinical decision supports for physicians. In recent advancements of intelligent medical and healthcare systems, Bates *et al.* [30] and Ammenwerth *et al.* [31] provided evaluations and commandments on modern healthcare information systems, and demonstrated that some well-built knowledge bases used in clinical decision supports and healthcare information systems can significantly promote the practices of evidence-based medicine and medical informatics. Hence, Wang *et al.* [32] applied a hybrid case-based reasoning approach to construct an efficient knowledge-based treatment planning system to provide early intervention in mental healthcare.

To fulfill the extensible, customizable, and configurable demands of the healthcare systems for patients with different health, age, and disease conditions under various rehabilitation and nursing environments, this study develops and integrates an efficient knowledge-based system and a component-based framework to design an intelligent and flexible home healthcare system. The major technical features of the proposed intelligent component-based home healthcare system framework are given as follows:

1. The knowledge-based system developed in this study integrates an efficient rule-based reasoning model and the flexible knowledge rules for efficiently and rapidly determining the necessary physiological and medication treatment procedures from the software modules, video camera sensors, communication devices, and physiological sensor information (such as body temperature, heart rate, blood pressure, blood oxygen values, breath meters, and ECGs).
2. This knowledge-based system offers high flexibility for improving and extending the system further to satisfy new patient and caregiver healthcare and monitoring demands by updating the knowledge rules in the inference mechanism.
3. The proposed component-based framework incorporates four major components: a real-time video-monitoring component, physiological information and treatment-monitoring module, monitoring information transmission module, and remote patient-monitoring component. All of these proposed functional components in this study are reusable, configurable, and extensible for system developers to design and implement customized home healthcare systems rapidly to meet various demands of patients and caregivers from ubiquitous healthcare monitoring aspects.

The implementation and experimental results indicate that the proposed intelligent homecare system framework can satisfy the extensible, customizable, and configurable demands of ubiquitous healthcare systems for different patients and caregivers under various rehabilitation and nursing conditions.

The rest of this study is organized as follows: Section 2 describes the overall framework and major components of the proposed component-based healthcare system. Section 3 presents the architecture and features of the knowledge-based system for home healthcare applications. The results and application scenarios are demonstrated in Section 4. Finally, Section 5 offers the conclusions to this study.

## 2. Component-Based Healthcare System Framework

The proposed component-based framework for intelligent homecare systems based on the UML modeling process [33,34]. To provide a flexible, convenient, and extensible homecare system for patients and caregivers, this study implemented the proposed homecare monitoring system based on an extensible component-based framework. This component-based framework is designed according to the following reusable modules:

1. The real-time video codec component integrates the proposed fast and real-time monitoring video coding and decoding technique to record computationally and effectively the monitoring videos of patient rehabilitation activities and behaviors at the patient's home with high visual quality and economic storage. Furthermore, the proposed codec component is portable for use on various devices and Web browsers, and implements a set of flexible software interfaces for recording and retrieving the videos to provide a convenient and rapid development of various applications specific to local and remote homecare monitoring systems.
2. The physiological information and treatment-monitoring module can measure and acquire patient physiological data using sensors (such as body temperature, heart rate, blood pressure, blood oxygen values, breath meters, and ECGs), and record the treatment plans according to physician prescriptions in an XML archiving database. The recorded physiological data and treatment plans can be retrieved and viewed conveniently by caregivers using various devices or Web browsers. The caregiver can also adopt this component to establish alert schedules according to the patient's treatment plans, as well as establish the alarm conditions for the patient's physiological warning situations. Based on the determination results of the patient's physiological and treatment situations provided by the proposed knowledge-based system, the patient can be automatically alerted to follow the treatment process, such as a medication schedule, and caregivers can immediately be alerted when the patient's physiological status worsens.
3. The monitoring information transmission module corresponds with the mentioned real-time video codec component and physiological information and treatment-monitoring module for transmitting patient-monitoring videos, physiological data, and treatment records to caregivers' remote handheld devices and Web browsers through the Internet and mobile communication networks. The transmission module involves using RTP (real-time transport protocol) to transmit streams of patient-monitoring videos while using the TCP/IP protocol to transmit patient physiological data, treatment records, and warning situations.
4. The remote patient-monitoring component supports remote patient-monitoring applications. This component, the real-time video codec component, and the physiological information and treatment-monitoring module are conveniently distributed and can be easily installed in the remote client-side handheld devices and Web browsers of caregivers. Thus, caregivers can request and obtain patient-monitoring video streams, physiological data, treatment records, and alert messages of warning states from the main health care monitoring system at a patient's home using mobile devices or Web browsers. These videos and data can then be displayed and retrieved by applying the client-side real-time video codec component and physiological information and treatment-monitoring component.

The overall system framework of the proposed homecare monitoring system is described using the scenario cases listed in Table 1, which also lists brief semantics for the scenario cases provided using the proposed system. The major components and modules of the proposed framework are accordingly described in the following subsections.

### 2.1. Real-Time Video Codec Component

To monitor and record a patient's rehabilitation in various home settings and provide convenient caregiver observation, the proposed real-time video codec component encapsulates the proposed fast and real-time monitoring video compression and decompression technique (introduced in the next subsection), and integrates and implements the crucial functions and software interfaces for video-monitoring applications used as software components. In coordinating with the main system application and other modules, the proposed real-time video codec component is responsible for providing convenient and portable interfaces to record and retrieve the patient's videos for different systems, mobile terminal devices, and Web browsers.

To meet the demands of monitoring and recording patient rehabilitation activities, efficient recording of real-time image sequences of patient activities in various environments is an essential mission. Therefore, the monitoring video core of the proposed real-time video codec component adopts a wavelet-based fast video compression technique [35] that can provide low computational costs, low memory consumption, and high visual quality of the compressed monitoring video frames. Most video-monitoring systems are applied to monitor specified areas continuously for an extended duration. Therefore, applying traditional, still-image compression methods, such as JPEG2000 [36,37], at all times wastes considerable storage space. Some conventional standard-motion image compression techniques, such as MPEG-4 [38,39], involve considerable computational complexity for simultaneously monitoring numerous monitoring areas. Most of the conventional approaches cannot meet the high frame-rate requirements of a multichannel video-monitoring system. Therefore, this study applied and integrated a simplified video compression method with a high compression ratio and speed to accommodate efficiently the requirements of patient-monitoring applications [35].

**Figure 1.**
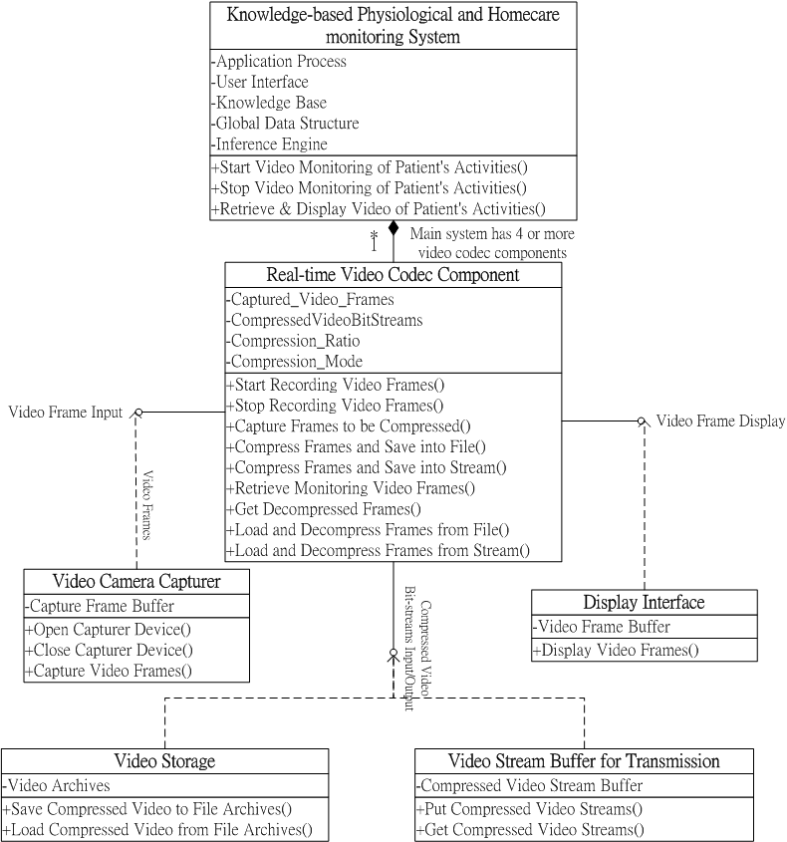
Class diagram of the real-time video codec component.

As illustrated in the UML class diagram of the proposed video codec component in Figure 1, three major software interfaces were implemented for conveniently supporting the functions of recording and retrieving patient monitoring videos: (1) video frame input; (2) compressed video bit-stream input/output; and (3) video frame display. First, the video frame input interface should provide functions acquiring video frames from the camera sensors mounted in monitoring regions, as well as compressing the acquired video frames into bit-streams. The compressed video bit-stream input/output interface provides a set of functions for storing and loading compressed monitoring video bit-streams into file archives (such as hard disks and cloud storages) and transmits and receives compressed video bit-streams from file archives or remote network transmission devices (such as Internet and mobile communication networks) by correspondence with the monitoring information transmission module. Accordingly, using a video frame display interface, the archived or remotely transmitted compressed video bit-streams can be decompressed and retrieved using the display devices of the local server-side system, client-side handheld devices, or Web browsers; thus, from anywhere at any time, caregivers can ubiquitously monitor the patient's care activities and behaviors using various remote devices from different places.

### 2.2. Physiological Sensing and Treatment-Monitoring Module

Assisting patients and caregivers in understanding health and rehabilitation conditions, regularly observing patients' physiological data records, and maintaining the treatment plans prescribed by physicians are all crucial for patients' rehabilitation processes. Therefore, the following are paramount for an intelligent patient healthcare monitoring system: conveniently sensing and recording physiological data (e.g., body temperature, heart rate, blood pressure, blood oxygen values, breath meters, and ECGs) from home physiological measurement sensors; establishing patients' warning conditions according to the determination of the knowledge-based system; alerting patients to follow rehabilitation treatment plans (such as medication schedules and recording the process); and alerting caregivers when patients' health statuses deteriorate according to the determined warning conditions by the knowledge-based physiological and homecare monitoring system.

To facilitate the application of the corresponding modules of the knowledge-based physiological and home care monitoring system, the physiological sensing and treatment-monitoring module is designed to provide portable and efficient interfaces to acquire and retrieve patient physiological data from sensors. The module also provides the interfaces to monitor the patients' treatment processes and physiological statuses and provide alerts using different server-side systems and remote mobile terminal devices. In addition, the module functions to determine the warning conditions of patient physiological statuses by using the knowledge-based system.

**Figure 2.**
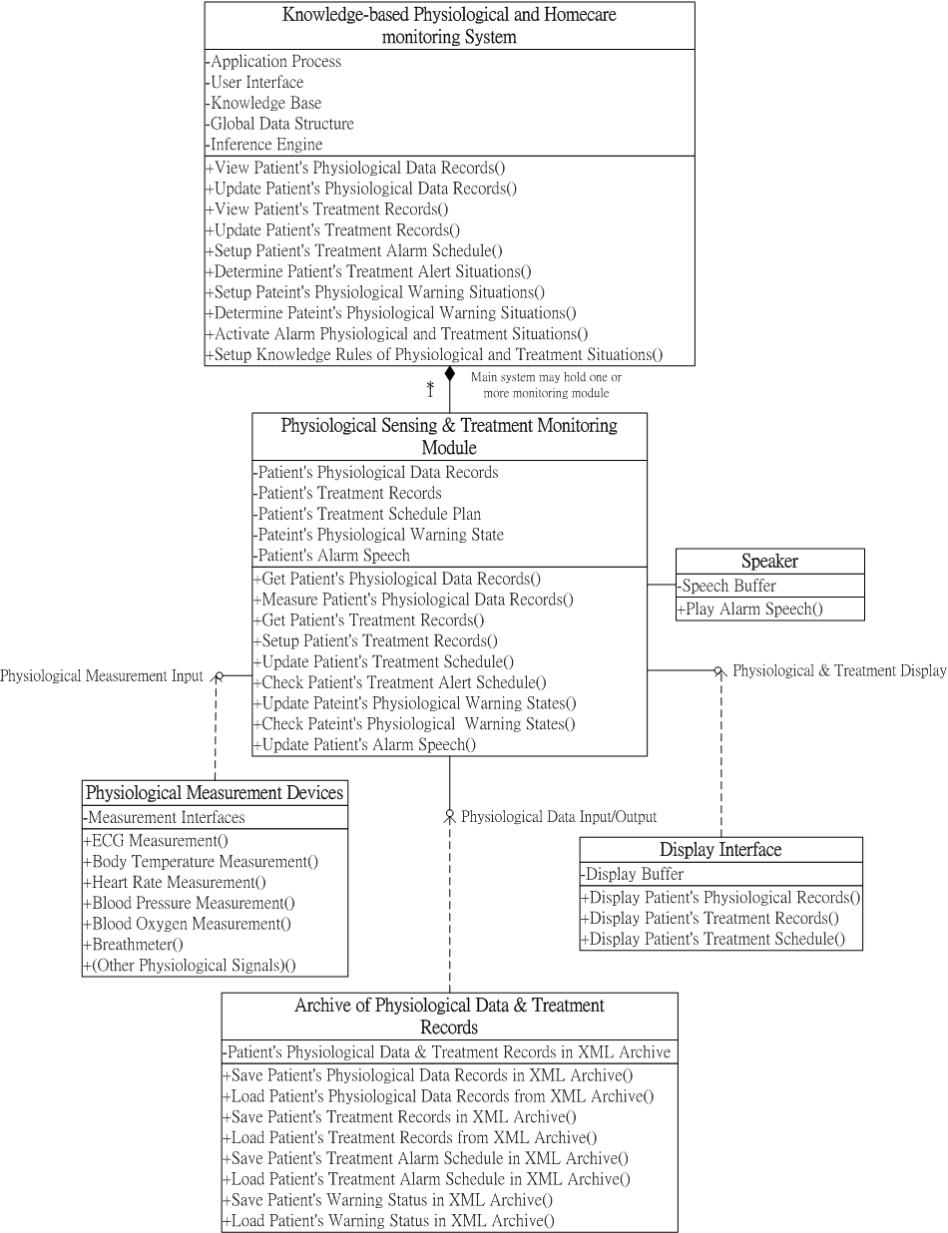
Class diagram of the physiological sensing and treatment-monitoring module.

Regarding the class diagram in Figure 2, the physiological sensing and treatment-monitoring module provides a set of crucial functions and three software interfaces for recording, monitoring, and alerting caregivers on patient physiological statuses and treatment processes according to the determination of the knowledge-based system: (1) physiological data input; (2) physiological data input/output; and (3) physiological and treatment display. The physiological data input interface supplies the functions for acquiring the patients' physiological data from domestic physiological measurement devices. Using the physiological data input/output interface, the acquired patient physiological data and treatment records of rehabilitations can be recorded in XML-formatted documents with timestamps. These data records can be stored in file archives (such as hard disks) or transmitted to communication devices for caregivers' remote observation through cooperated correspondence with the monitoring information transmission module. The archived patients' physiological data and treatment records can also be retrieved and observed by caregivers through the local server-side archives, as well as remote handheld devices or Web browsers.

To alert patients to take medicine according to their treatment plans, caregivers and patients can establish alert schedules and corresponding alarm speeches to automatically inform patients using the corresponding functions provided by the module. Moreover, caregivers and patients can also establish patient physiological warning states by using the provided functions, so that caregivers can promptly receive the alarm notifications on their remote monitoring devices once the patients' health conditions, for example, suddenly deteriorate. For retrieving and displaying the archived patients' physiological data and treatment records, the physiological and treatment displays provide convenient functions for parsing and reformatting the XML archives of the patients' physiological data and treatment records into viewable contents, to offer caregivers ubiquitous retrieval and monitoring of patient rehabilitation processes from anywhere at any time.

### 2.3. Monitoring Information Transmission Module

For transmitting the patients' monitoring videos as well as health and rehabilitation conditions to caregivers' remote browsers and monitoring devices, or obtaining patients' monitoring data from the main knowledge-based physiological and homecare monitoring system at a patient's home, the proposed monitoring information transmission module is designed to correspond with the real-time video codec component and the physiological information and treatment-monitoring module mentioned in the previous subsections. To transmit the patients' monitoring data efficiently and flexibly between caregivers' remote browsers and handheld devices and the main monitoring system in patients' homes, the proposed transmission component implements and integrates the RTP (real-time transport protocol) for transmitting the monitoring video records, as well as the TCP/IP protocol for transmitting health and rehabilitation records. Therefore, the transmission component is designed to provide flexible and portable interfaces and functions to correspond with the applications of the knowledge-based physiological and home care monitoring system, real-time video codec component, and physiological information and treatment-monitoring module. The transmission component then transmits the corresponding monitoring video streams, physiological data, and treatment conditions to remote monitoring devices through the Internet or wireless communication networks.

**Figure 3.**
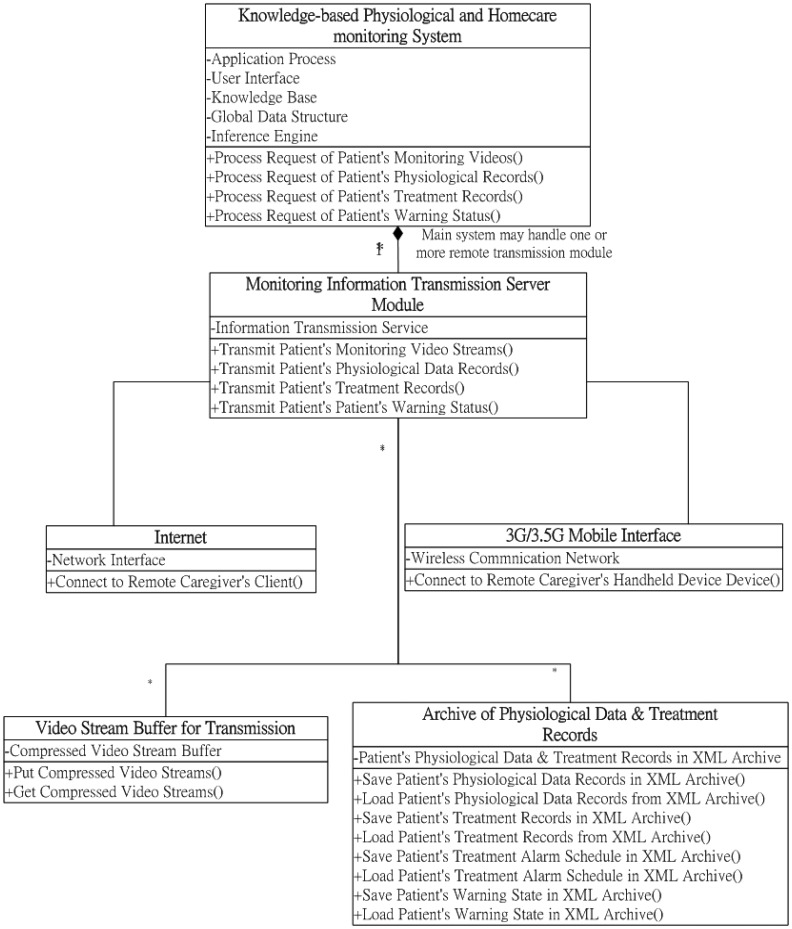
Class diagram of the monitoring information transmission module.

As depicted in the class diagram of the proposed transmission module in Figure 3, three major functions are provided to transmit the patients' monitoring data to caregivers' remote browsers and monitoring devices: (1) transmitting the patients' monitoring video streams; (2) transmitting the patients' physiological data records; (3) transmitting the patients' treatment records; and (4) transmitting the patients' warning statuses. Using the function of transmitting the patients' monitoring video streams, the transmission component transmits the compressed monitoring video bit-streams (which have already been inputted in the video stream buffer using the real-time video codec component) to the remote terminal devices through the Internet or mobile communication network devices through the RTP transmission protocol. Regarding the transmission process applications of the archived patients' physiological data or treatment records, the transmission component first loads the desired patients' physiological data or treatment records from the archive device, and then transmits these records to the remote terminal devices through the Internet or mobile communication network devices through the TCP/IP protocol. Similarly, when caregivers must be informed of patients' warning statuses, using the transmitting function of the transmission component, then the alert notification message is transmitted to the caregivers' remote devices through the Internet or handheld personal communication network devices.

### 2.4. Remote Patient Care and Monitoring Component

**Figure 4.**
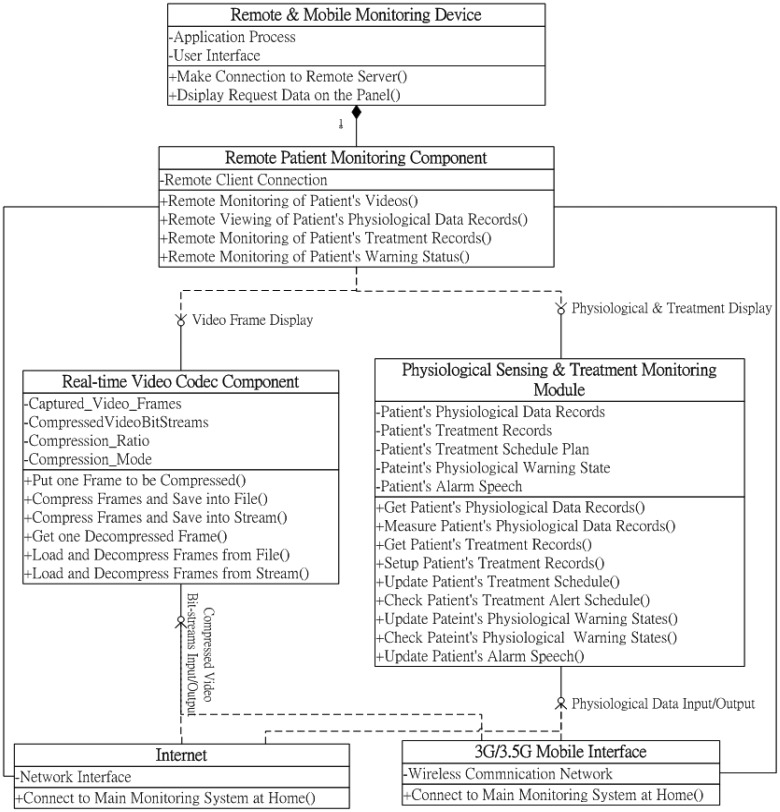
Class diagram of the remote patient care and monitoring component.

The three mentioned components can also be easily distributed for developing remote monitoring applications to be installed and operated on caregivers' remote handheld devices or browsers. Accordingly, the proposed remote patient care and monitoring component primarily provides the required functional interfaces for caregivers to request and retrieve patient-monitoring video streams, physiological data, and treatment records. As illustrated in the class diagram of the remote monitoring component in Figure 4, three major functions are designed for remotely requesting and retrieving the patient's monitoring data through caregivers' remote mobile devices or browsers: (1) remote monitoring of patient videos; (2) remote viewing of patient physiological data records; (3) remote monitoring of patient treatment records; and (4) remote monitoring of patient warning status. Using the remote monitoring function of patient videos, the compressed monitoring video bit-streams, which are transmitted from the main homecare system at a particular patient's home, can be acquired from the Internet or mobile communication network devices via the RTP protocol, and then the video bit-streams can be decompressed and displayed on caregivers' mobile devices or browsers with the client-side real-time video codec component. By contrast, when using the remote monitoring component, patient physiological data records archived in the main homecare system can be retrieved using the remote viewing function of patient physiological data records through network communication devices via the TCP/IP protocol. Accordingly, patient treatment records can be obtained similarly using the remote monitoring function of patient treatment records. The retrieved patients' physiological data and treatment records in XML-archived format are then parsed and displayed on caregivers' mobile devices or browsers using the client-side physiological information and treatment-monitoring component. Moreover, when the main homecare system sends an alert message of a particular patient's warning status to caregivers, by applying the remote monitoring function of the patient's warning status, the remote monitoring component acquires the alert message from network communication devices via the TCP/IP protocol, and then the information of the patient's physiological warning status is displayed on a caregiver's handheld device or browser using the client-side physiological information and treatment-monitoring component.

### 2.5. Overall Component-Based Homecare System Framework

**Figure 5.**
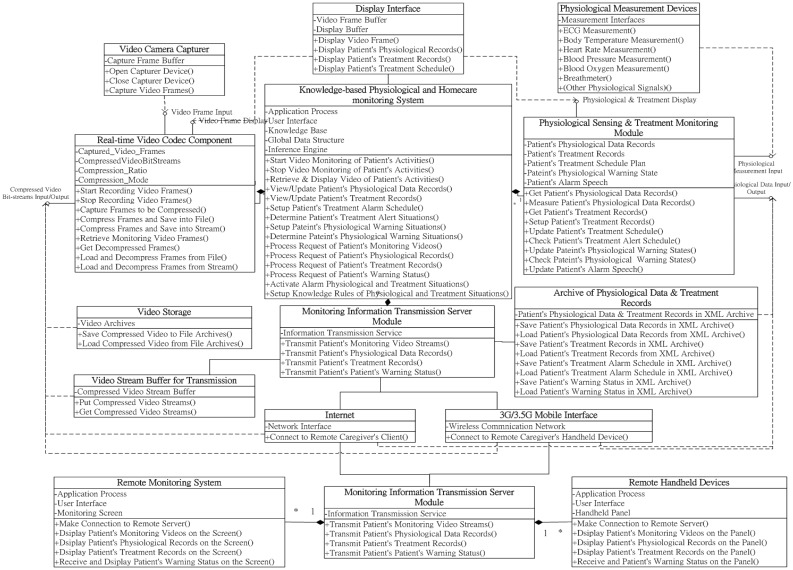
Integrated class diagram of the proposed component-based home care system framework.

Based on the mentioned uses and functionalities of the components and modules of the proposed component-based design framework, the design framework of an intelligent home care system can be accomplished by integrating these components and modules. As the complete UML class diagram shows in Figure 5, the connection and correspondence of the components and modules for designing a flexible intelligent homecare system is comprehensible for system developers in implementing and maintaining customized homecare systems. All software components and modules in this component-based design framework are reusable, replaceable, and extensible for system developers to establish customized intelligent knowledge-based homecare systems to meet different patient healthcare monitoring demands. For instance, using different numbers of cameras and physiological sensor devices for monitoring various numbers of patients of different ages with different diseases can enable customizing the healthcare systems by changing the configurations of the real-time video codec components and physiological information and treatment-monitoring modules.

## 3. Knowledge-Based Physiological and Homecare Monitoring System

**Figure 6.**
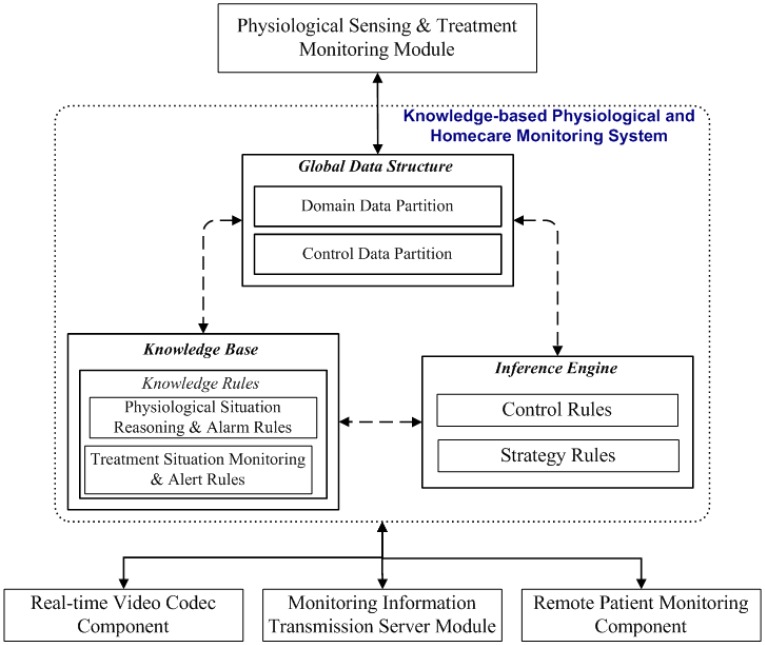
Proposed knowledge-based physiological and homecare monitoring system.

The knowledge-based physiological and homecare monitoring system of the proposed homecare framework can monitor and determine patients' physiological situations by interacting with real-time video codec components, physiological sensing and treatment-monitoring modules, monitoring information transmission modules, and remote patient-monitoring components. This knowledge-based system is constructed according to the mentioned modules, a partitioned global data structure, and a rule-based reasoning system, as Figure 6 shows.

### 3.1. Knowledge-Based System

As depicted in Figure 6, the proposed knowledge-based system adopts a three-level rule-based reasoning model, which comprises knowledge, control, and strategy rules, for determining efficiently and rapidly the necessary physiological and medication treatment procedures from the software modules and physiological sensor information (such as body temperature, heart rate, blood pressure, blood oxygen values, breath meters, and ECGs). This knowledge-based system can provide high flexibility to improve and extend the system further by updating the knowledge rules in the inference mechanism. The knowledge rules of the proposed system are divided into two sets, physiological condition determination rules and medication action monitoring rules, which encode typical physiological signal features and medication procedures according to most patients' physiological characteristics and therapies.

The control rules, which are composed of an inference engine, then determine which physiological features and medication procedures to analyze and evaluate, as well as which subsequent home care process to perform and which events to activate. These control rules include two categories: the focus-of-attention rules and the meta-rules. The focus-of-attention rules determine the next physiological features and medication procedures to evaluate the use of the knowledge rules, whereas the meta-rules determine the processing phases and feature configurations to specify the set of knowledge rules to perform next. The strategy rules decide the invocation process of a particular set of control rules and determine their execution order on the physiological features and medication procedures.

To facilitate the rule-based reasoning process in analyzing and activating healthcare events, the proposed method adopts a global data structure containing domain and control data partitions to maintain the critical processing information of the processed physiological features and medication procedures, as well as immediate control statuses. The domain data partition includes features and information on the physiological features and medication procedures from the software modules and physiological sensor information to be processed by the knowledge-based physiological and homecare monitoring modules. The control data partition includes control information on the statuses of the extraction and identification processes and detailed records on any results maintained in the global data structure.

### 3.2. Knowledge-Based Physiological and Homecare Monitoring Process

The domain data of the textual components used in the knowledge-based physiological and homecare monitoring process can be categorized into two types:

1. *Patient physiological features*: These features are patients' physiological data obtained from physiological sensors in the *physiological measurement procedure* at a given time *t*.
2. Patient medication and treatment features: These features reveal the patients' medication and treatment procedures at a given time t.

First, the patients' physiological features are defined as follows:

- The heart rate of the patient measured at time *t*, as denoted by *H*_t_ and its unit is in beats per minute.
- The patient's body temperature measured at time *t*, as denoted by *T_t_*, and its unit in degrees Celsius.
- The patient's blood pressure value acquired at time *t*, as denoted by *P_t_*, and its unit is mmHg.
- The blood oxygen saturation value of the patient measured at time *t*, as denoted by *O_t_*, and its unit is the saturation ratio.
- The breath meter of the patient measured at time *t*, as denoted by *B_t_*, and its unit is times per minute.
- The ECG features of the patient measured at time *t*, as denoted by *C_t_*.

Second, the feature data used for patient medication and treatment features include the following:

- The current action frequency in this day, the total required number of physiological measurements per day, the most recent care time, and the required time interval of physiological measurements for a specified patient, as denoted by *PT*, *PTD*, *PL*, and *PI*, respectively.
- The present conducting frequency, the total required number per day, the most recent care time, and the corresponding time interval of medications required for a particular patient, as denoted by *MT, MTD, ML*, and *MI*, respectively.
- The current number of times, the total number required per day, the most recent care time, and the time interval of hygiene cares needed for a patient, as denoted by *HT*, *HTD*, *HL*, and *HI*, respectively.
- The present number of times, the total number required per day, the most recent care time, and the time interval of feeding required for a patient, as denoted by *FT*, *FTD*, *FL*, and *FI*, respectively.

For offering the patients' healthcare conditions and events, the following *physiological and treatment situations* involved by the knowledge rules, the corresponding modules, and the mentioned features are listed as follows:

1. **Normal situation**: The patient's physiological records exhibit favorable health conditions, and the patient is cared for according to the medication and treatment plans.
2. **Abnormal heart alarm**: The patient's heart rate, blood pressure, or ECG reveal abnormal features that might be caused by heart diseases. The system then alerts the doctors and caregivers to conduct further diagnoses and care procedures.
3. **Abnormal cardiorespiratory alarm**: The patient's breath rate or blood oxygen saturation value reflects abnormal conditions, indicating that the patient's physiological cardiorespiratory conditions may deteriorate. The doctors and caregivers are then alerted to perform diagnoses and care procedures.
4. **Abnormal body temperature alarm**: The patient's body temperature reveals some abnormal values, reflecting that the patient's physiological body conditions may deteriorate. The system then alerts the doctors and caregivers to conduct further diagnoses and healing procedures.
5. **Irregular physiological measurement alert:** The patient misses some regular physiological measurement procedures after a particular period according to his/her treatment plan. The patient and caregivers are then alerted to complete the regular physiological measurement procedures.
6. **Uncharacteristic medication alert:** The patient neglects some expected medication procedures (such as taking medication) after a period according to his/her treatment plan. The system then alerts the patient and caregivers to complete the patient's required medication procedures.
7. **Hygiene alert:** Some expected hygiene procedures of the patient are passed over after a particular period. The patient and caregivers are then alerted by the system to accomplish the required hygiene procedures.
8. **Feeding alert:** Some required feeding procedures for the patient are not performed for a particular period. The system then alerts the patient and caregivers to address the nutritional needs of the patient.

The *control rules* and *strategy rules* for the inference engine to determine the mentioned physiological and treatment situations are listed as follows.

#### Control rule (C-1)

IF:

The status is a *normal situation* and no alarm occurs.

THEN:

1. Set the current process to the patient medication and treatment determination.
2. Apply the patient medication and treatment determination rules on the patient's medication and treatment features measured.

#### Control rule (C-2)

IF:

- The patient medication and treatment determination process has been regularly performed.
- The physiological measurement procedures of the patient have been regularly performed.

THEN:

1. Set the current process to the *physiological situation reasoning*.
2. Apply the physiological situation reasoning rules to the patient's physiological features measured from sensors.

#### Strategy rule (S-1)

IF:

There are new physiological features, treatment, or medication events being obtained from the corresponding sensors and components.

THEN:

Apply all of the control rules of the physiological situation reasoning and patient medication and treatment determination processes to determine the patients' possible physiological and treatment situations.

The knowledge rules for the physiological situation reasoning process, which are adopted for determining the patients' possible body and physiological conditions and alerting doctors and caregivers for necessary further diagnosis and healing procedures, are described as follows:

#### Knowledge rule (K-1)

IF:

- The current process is physiological situation reasoning.
- The patient's current measured heart rate *H_t_* is higher than a given upper criterion (e.g., *H_t_* > 100 times per minute), or lower than a given lower criterion (e.g., *H_t_* < 60 times per minute).
- Otherwise, if the patient's current blood pressure value *P_t_* is higher than a given upper criterion (e.g., *P_t_* > 160 mmHg), or lower than a given lower criterion (e.g., *P_t_* < 90 mmHg).
- Otherwise, if the patient's current ECG *C_t_* reflects abnormal magnitudes and cyclic patterns as compared to those of normal situations.

THEN:

1. It is inferred that the patient's body conditions deteriorate because of possible heart diseases; the system then activates the *abnormal heart alarm situation*.
2. The system alerts caregivers and doctors by using remote and handheld devices with the corresponding information transmission module and remote patient-monitoring component as mentioned in the previous section.
3. The patient's heart rate, blood pressure, and ECG information, as well as real-time monitoring videos (obtained from the physiological information and treatment monitoring module and real-time video codec component) are sent to caregivers and doctors through the monitoring information transmission module and the remote patient-monitoring component, to facilitate further diagnosis and necessary healing procedures.

#### Knowledge rule (K-2)

IF:

- The status is an abnormal heart alarm situation.
- After performing doctors' and caregivers' diagnosis and healing procedures on the patient, the patient's current measured heart rate *H_t_* returned to reference values (e.g., 60 < *H_t_* < 100), and blood pressure value *P_t_* recovered to reference values (e.g., 90 < *P_t_* < 160), as well as the current ECG *C_t_* recovered to regular magnitudes and cyclic patterns.

THEN:

The patient's physical condition is recovered, the alarm is suspended, and the situation is set as a normal condition.

#### Knowledge rule (K-3)

IF:

- The current process is physiological situation reasoning.
- The patient's current measured breath meter *B_t_* is higher than a given upper criterion (e.g., *B_t_* > 24 times per minute) or lower than a given lower criterion (e.g., *B_t_* < 8 times per minute).
- Otherwise, if the patient's current blood oxygen saturation *O_t_* becomes lower than a given lower criterion (e.g., *O_t_* < 90%).

THEN:

1. It is inferred that the patient's breathing conditions are deteriorating because of possible respiratory disease. The system then activates the *abnormal cardiorespiratory alarm situation*.
2. The system alerts caregivers and doctors through remote and handheld devices, the monitoring information transmission module, and the remote patient-monitoring component.
3. The patient's breath rates and blood oxygen saturation values, as well as real-time monitoring videos are transmitted to the caregivers and doctors through the information transmission module and the remote monitoring component, to facilitate further diagnosis and healing procedures in cardiorespiratory recovery.

#### Knowledge rule (K-4)

IF:

- The status is an abnormal cardiorespiratory alarm situation.
- After performing doctors' and caregivers' diagnosis and healing procedures on the patient, the patient's current measured breath rate *B_t_* recovers to reference values (e.g., 8 < *B_t_* < 24) and blood oxygen saturation *O_t_* returns to normal values (e.g., *O_t_* > 90%).

THEN:

The patient's body condition is temporarily recovered, the alarm is suspended, and the situation is set as the normal condition.

#### Knowledge rule (K-5)

IF:

- The current process is physiological situation reasoning.
- The patient's current acquired body temperature *T_t_*, is higher than a given upper criterion (e.g., *T_t_* > 37 °C) or lower than a given lower criterion (e.g., *T_t_* < 35 °C).

THEN:

1. It is determined that the patient's bodily conditions may succumb to illness because of possible colds or flues. The system then activates the *abnormal body temperature alarm situation*.
2. The system alerts caregivers and doctors through remote and handheld devices, the monitoring information transmission module, and the remote patient-monitoring component.
3. The patient's body temperature information and real-time monitoring videos are transmitted to the caregivers and doctors through the monitoring information transmission module and the remote patient-monitoring component, to notify them to perform further diagnosis and healing procedures.

#### Knowledge rule (K-6)

IF:

- The status is an abnormal body temperature alarm situation.
- After performing doctors' and caregivers' diagnosis and procedures on the patient, the patient's current measured body temperature returns to reference values (e.g., 35 < *T_t_* < 37).

THEN:

The patient's physical condition temporarily recovers, the alarm is suspended, and the situation is set as the normal condition.

The patient medication and treatment determination rules for evaluating and monitoring the regularity and appropriateness of patients' medication and treatment procedures are given as follows.

#### Knowledge rule (K-7)

IF:

- The current process is patient medication and treatment determination.
- The current number of times *PT* of performing the physiological measurement procedures do not meet the daily required times *PTD* (*i.e.*, *PT* < *PTD*).
- The current observed time *t* to the most recent physiological measurement time *PL* has already elapsed over the required time interval (*i.e.*, (*t* – *PL*) > *PI*).

THEN:

1. It is inferred that the patient and his/her caregivers missed required physiological measurement procedures prescribed in the treatment plan. The system then activates the *irregular physiological measurement alert* situation.
2. The system alerts caregivers and sends the patient's recent physiological measurement records and real-time monitoring videos to the caregivers' remote and handheld devices through the monitoring information transmission module and remote patient-monitoring component, to notify them to perform the required physiological measurement procedures for the patient.

#### Knowledge rule (K-8)

IF:

- The current process is patient medication and treatment determination.
- The current number of times *MT* of the patient's medication procedures do not meet the daily required times *MTD* (*i.e.*, *MT* < *MTD*).
- The current evaluated time *t* of the patient's most recent medication time *ML* has passed the required medication time interval (*i.e.*, (*t* – *ML*) > *MI*).

THEN:

1. It is determined that the patient missed the required medication as prescribed in the treatment plan. The system then activates the *uncharacteristic medication alert* situation.
2. The system alerts the caregivers and sends the patient's recent medication records and real-time monitoring videos to the caregivers' remote and handheld devices through the monitoring information transmission module and remote patient-monitoring component, to alert them to administer the required medication to the patient.

The knowledge rules for the *patient medication* and *treatment determination* process that are applied to determine the patients' possible treatment and medication situations in rehabilitation, and alerting the caregivers for necessary care procedures, are given as follows.

#### Knowledge rule (K-9)

IF:

- The current process is patient medication and treatment determination.
- The current number of times HT of performing hygiene cares for the patient do not satisfy the daily required times HTD (i.e., HT < HTD).
- The current examined time t to the patient's most recent hygiene care time HL has passed the required hygiene action time interval (i.e., (t – HL) > HI).

THEN:

1. It is determined that the patient and his/her caregivers neglected to perform the needed hygiene care in the rehabilitation process. The system then activates the *hygiene alert* situation.
2. The system alerts the caregivers and sends the patient's recent hygiene care records and real-time monitoring videos to the caregivers' remote and handheld devices to inform them to conduct the required hygiene procedures for the patient.

#### Knowledge rule (K-10)

IF:

- The current process is patient medication and treatment determination.
- The current number of times *FT* of feeding for the patient do not satisfy the daily required times *FTD* (*i.e.*, *FT* < *FTD*).
- The current monitored time *t* to the patient's last feeding time *FL* missed over the required feeding time interval (*i.e.*, (*t* – *FL*) > *FI*).

THEN:

(1) It is determined that the patient and his/her caregivers forwent necessary feeding in the rehabilitation process. The system then activates the *feeding alert* situation.

(2) The system alerts the caregivers and sends the patient's recent feeding records and real-time monitoring videos to the caregivers' remote and handheld devices to remind them to conduct the required feeding procedures for the patient.

Similar to the recovery knowledge rules for the physiological situation reasoning processes, the recovery rules can be implemented for determining whether the patient has received the required medication and treatment after caregivers' perceiving the alert situations of physiological measurement, medication, hygiene, and feeding according to knowledge rules K-7 to K-10. The system then suspends the mentioned alerts and reverts the system to the normal condition.

## 4. Results

**Figure 7.**
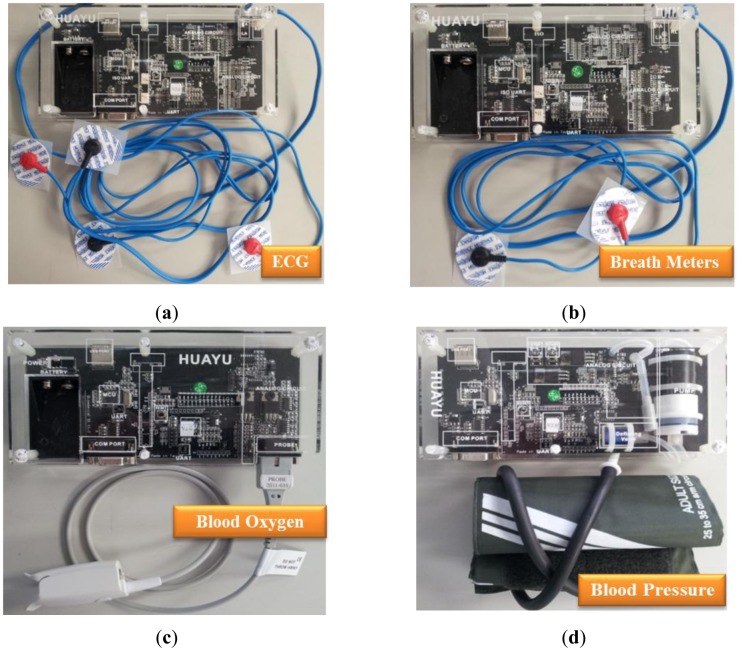
Embedded platform and physiological sensor modules of the proposed homecare system. (**a**) ECG sensor module. (**b**) Breath meter sensing module. (**c**) Blood oxygen sensor module. (**d**) Blood pressure sensor module.

**Figure 8.**
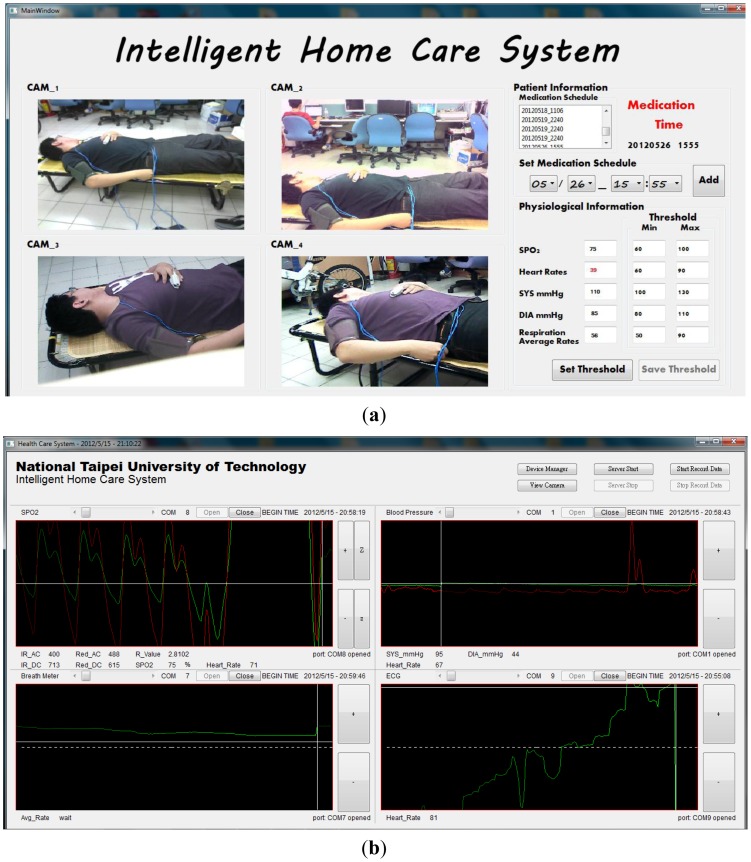
Prototype system implemented by the proposed intelligent component-based homecare system. (**a**) Main screen of the prototype homecare system. (**b**) Operation screen of measuring and viewing the patients' physiological data records.

**Figure 9.**
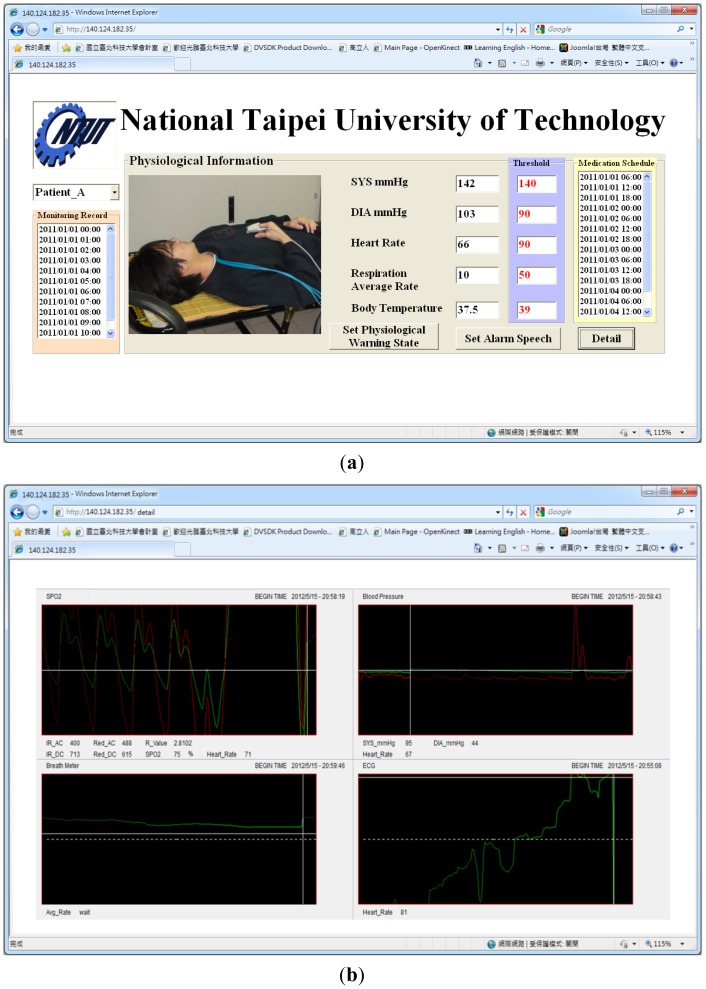
Remote monitoring interfaces on the Web browsers. (**a**) Main remote monitoring interface on the Web browser. (**b**) Remote monitoring screen of the detailed physiological data on the Web browser.

**Figure 10.**
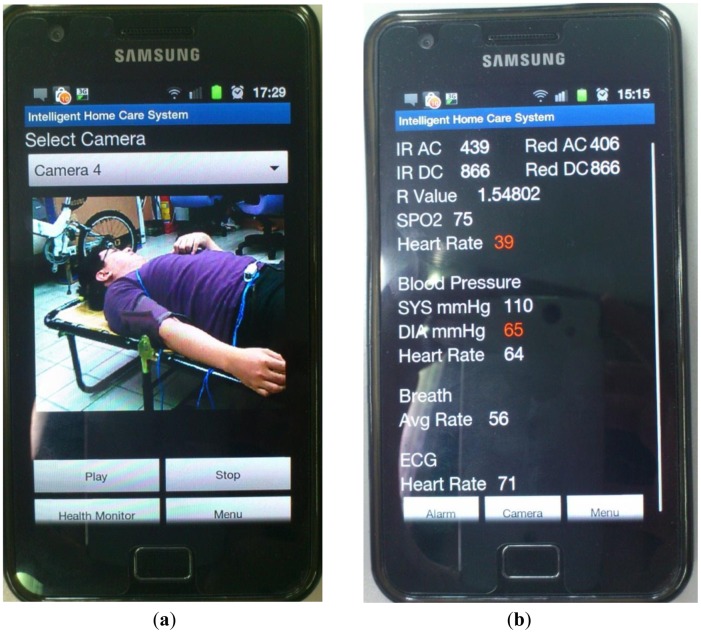
Remote monitoring interfaces of the handheld devices. (**a**) Main remote monitoring interface on the handheld device. (**b**) Example of the patient's physiological warning message shown on the handheld device.

The prototype home healthcare system, as shown in Figures 7–10, was implemented based on the proposed intelligent knowledge-based system module and the component-based framework with integration of the real-time video codec components, physiological information and treatment-monitoring component, monitoring information transmission module, and remote patient-monitoring components. This prototype implementation of the home healthcare system integrated four CCD cameras to facilitate the monitoring of four separate video-monitoring settings, measuring patient physiological data (such as body temperature, heart rate, blood pressure, blood oxygen values, breath meters, and ECGs) using measurement devices, and transmitting the videos, physiological data, treatment records, and warning messages to wireless and handheld devices.

In this study, the main system of the proposed prototype intelligent homecare system was implemented on an ARM-based embedded platform, the Marvell PXA310 platform. The TI Marvell PXA310 is an efficient economical solution for portable and handheld systems from the ARM processor family. This platform consists of one Marvell PXA310 ARM-based general-purpose processor with a 624-MHz operational speed to implement the software modules, knowledge-based system, and physiological sensor data. This platform also includes 256 MB of flash ROM memory for storing the embedded Linux OS kernel, Qt-based GUI system, and the proposed homecare software modules, and 128 MB of DDR memory for executing the software modules. Regarding to the archiving aspect of the monitoring video records, this embedded prototype system record and compress the patient monitoring videos in CIF format (352 × 288 pixels) with true color (three bytes per pixel) and real-time (30 frames per second) acquired from the cameras. In addition to the main platform and physiological sensors, the peripheral devices such as video-sensing devices, wireless network communication module, and other in-vehicle control devices are also integrated to accomplish an embedded intelligent homecare system. The smart handheld devices are also adopted for the caregivers to ubiquitously monitor the patients' rehabilitation conditions, each of which consist of a dual-core Samsung Cortex-A9 ARM-based processor with an 1.2-GHz computational frequency and 1 GB of DDR memory for performing the remote patient monitoring components. The embedded platform and physiological sensor modules of the proposed embedded intelligent homecare system are shown in Figure 7. The remote monitoring screen connected to the proposed embedded intelligent homecare system is shown in Figure 8.

The proposed intelligent homecare system can provide the following functions and features:

1. **Video monitoring of patients' activities**: The system can monitor and record videos of rehabilitation situations and behavior of the patient using multiple CCD cameras, and the videos can be accordingly stored in archives. As shown in the main operating screen depicted in Figure 8(a), the home healthcare system can simultaneously monitor four separate settings of patients' activities, record the patients' basic physiological data, warning statuses, and treatment, as well as provide the archived monitoring videos and physiological and treatment records for patients and their caregivers' retrieval.
2. **Monitoring of Patients' physiological data and treatment records:** The system can record patients' physiological data records and corresponding treatment plans (such as medication schedules), and these records can be stored in an XML (extensible markup language) archiving database for caregiver review. In addition, at medication times or other scheduled healing procedures listed on the given treatment plan, the system can automatically alert the patient and record treatment details.
  As shown in the operation screen in Figure 8(b), the home healthcare system can provide advanced operational functionalities to measure and display the patient's physiological signals, including ECG data, blood pressure, blood oxygen values, and breath meters, while establishing alert mechanisms for the patients' physiological warning states according to their critical values of physiological signals, and then record these physiological signal records for further retrieval and observation by patients and caregivers.
3. **Ubiquitous monitoring of patients' monitoring videos:** From anywhere at any time, the patient's caregivers and family members can monitor the videos and physiological records of the patient's rehabilitation situations using handheld mobile devices via the Internet or wireless communication networks by installing portable software components of remote monitoring functions.
  Regarding the remote monitoring functions of patient healthcare and rehabilitation conditions, caregivers can use the Web browsers (as depicted in Figure 9(a)) or handheld devices (as depicted in Figure 10(a)) from anywhere at any time to monitor and retrieve the patients' videos, physiological data, and treatment records.
4. **Ubiquitous monitoring of patients' physiological and treatment conditions:** Caregivers and patients can install alert mechanisms for patient physiological warning states. When the measured physiological data indicate that a patient's physiological state is suddenly deteriorating, the proposed knowledge-based system immediately determines the possible physiological situations, and then alerts caregivers by sending notification messages to their remote handheld devices or Web browsers. Furthermore, if the patient misses any required treatment procedures for the rehabilitation processes, then the proposed knowledge-based system determines the missed treatment procedures and alerts the patient and caregivers to complete the regular physiological measurement procedures.
  Patient physiological data, including heart rate, body temperature, blood pressure, blood oxygen values, breath meters, and ECGs, can also be conveniently evaluated using the remote physiological data display interfaces, as shown in Figure 9(b). If patients' physiological states unexpectedly worsen as determined by the knowledge-based system, caregivers can receive notification messages through their handheld devices to notify them to conduct further diagnosis and healing procedures, as illustrated in Figure 10(b).

Based on the mentioned functions provided by the proposed intelligent homecare system, the ubiquitous healthcare services can be achieved feasibly and effectively in the following application scenarios:

### 4.1. Sample Scenario 1. Ubiquitous Monitoring and Alerting of Patients' Physiological Situations

**Figure 11.**
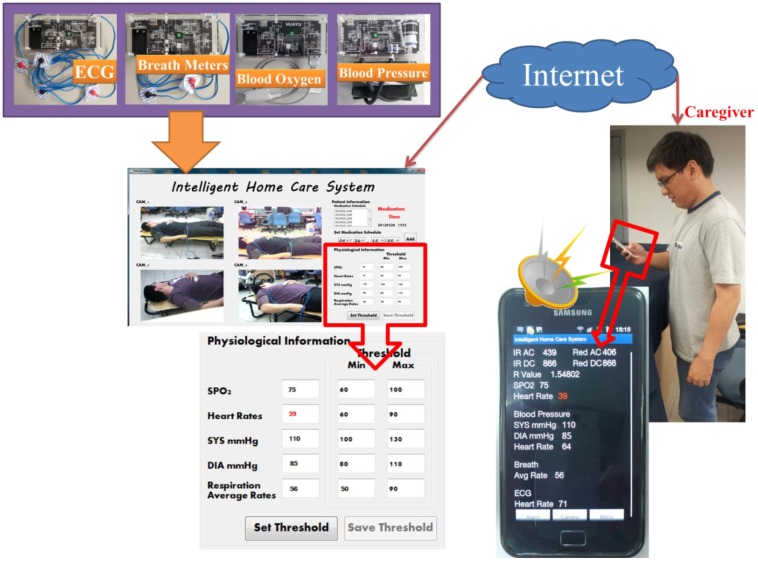
Illustrations of the ubiquitous monitoring and alerting of patients' physiological situations scenario.

The scenario of ubiquitous monitoring and alerting of patients' physiological conditions provided by the proposed intelligent homecare system is shown in Figure 11. Here, the caregiver can set the alerting thresholds for the patient's physiological states, including heart rate, blood pressure, blood oxygen values, breath meters, and ECG data, obtained from the corresponding modules. Once the patient's physiological states encounter any abnormal conditions (such as heart disease, abnormal cardiorespiratory, or body temperature conditions) as determined by the proposed knowledge-based monitoring system, then the caregivers and doctors immediately receive the patient's corresponding alarms, physiological data, and real-time monitoring videos through their handheld devices. As shown in the sample scenario in Figure 11, when the intelligent homecare system detects a patient's heart rate at lower than 60 beats per minute, the caregiver immediately receives the abnormal heart alarm, as well as the patient's heart rate, blood pressure, ECG information, and real-time monitoring videos to perform further diagnosis and care procedures.

### 4.2. Sample Scenario 2. Ubiquitous Monitoring and Alerting of Patients' Treatment Situations

**Figure 12.**
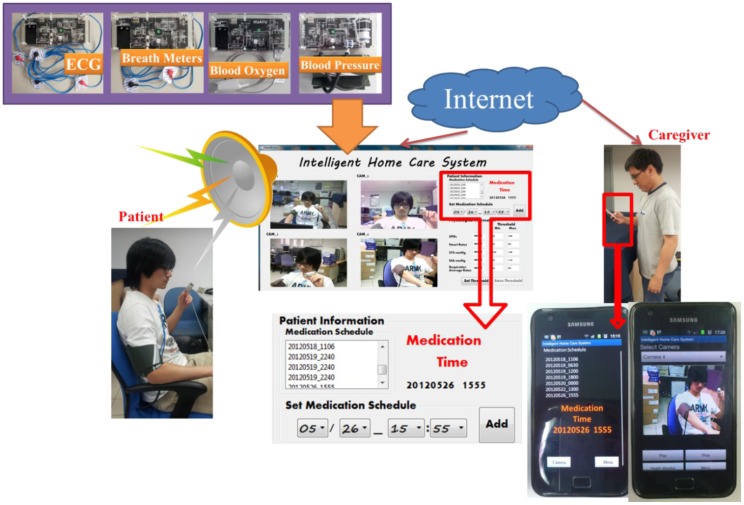
Illustrations of the ubiquitous monitoring and alerting of patients' medication scenario.

Regarding the application scenario depicted in Figure 12, the proposed intelligent home care system can provide ubiquitous monitoring and alerting of patient treatment processes (including regular physiological measurement, medication, hygiene, and feeding procedures) for both patients and caregivers. In this application scenario, the patient recuperates at home and the caregiver establishes the medication schedule using the main home care system. The medication procedures of the patient are then accordingly recorded and monitored. If a patient misses the required medication after a particular period, then the knowledge-based system activates the uncharacteristic medication alert situation and alerts the patient and caregivers by sending the patient's recent feeding records and real-time monitoring videos to the caregivers' handheld devices. Thus, caregivers can monitor and check the patients' care situations from any location using their handheld devices.

### 4.3. Sample Scenario 3. Ubiquitous Video Monitoring of Patients' Care Situations

**Figure 13.**
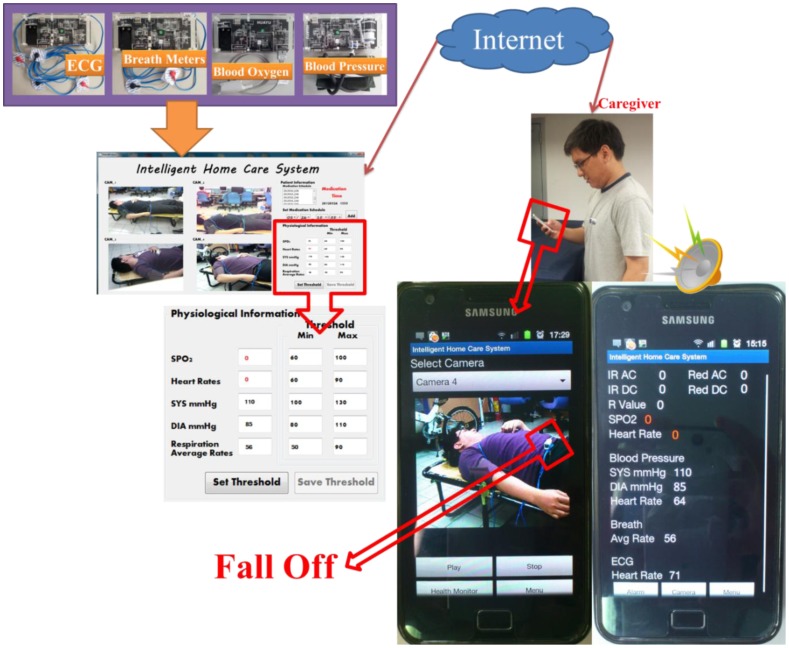
Illustrations of the ubiquitous video monitoring of patients' care scenario.

Regarding the application scenario shown in Figure 13, the proposed intelligent homecare system can provide physiological states and alarms, as well as offer ubiquitous video-monitoring functions for caregivers to perform immediate cares for patients. Caregivers can view a patient's monitoring videos to understand the patient's body conditions according to his/her appearance, and whether the patient's home care activities are appropriately conducted, to facilitate determining the healthcare and bodily conditions of the patient. In the scenario shown in Figure 13, the patient's physiological measurement sensors of heart rate and blood oxygen values unexpectedly fall, and the caregiver immediately receives the alarm of abnormal cardiorespiratory alarm situation of the patient through the handheld devices. The caregiver can instantaneously view the real-time monitoring videos of the patient's appearance and then determine whether the alarm is caused by the imprecise placements of the patient's physiological measurement modules. Therefore, the caregiver can help the patient restore the physiological sensors and the rehabilitation processes.

To evaluate the physiological and treatment alarm determination performance of the proposed homecare system, this study implemented and evaluated the home caring experiments on five patients for a period of one week. In our experiments, manually analyzing the patients' alarming situations obtain that there are 25 abnormal physiological alarming situations and 23 treatment alerting situations appeared on the five patients during the experimental period. Table 2 demonstrates that the proposed knowledge-based homecare system can accurately detect all the patients' abnormal physiological and treatment alarm situations, and notify the caregivers or doctors to provide necessary caring or healing actions via the remote and ubiquitous devices. Moreover, in our tests, the alarming information and monitoring videos can be timely received and displayed on the caregivers or doctors' handheld devices in a networking delay of less than 0.1 s after the patients' warning situations determined by the proposed homecare system.

## 5. Conclusions

This study proposes an efficient knowledge-based system and a component-based framework to design an intelligent and flexible home healthcare system for fulfilling the demands of extensibility, flexibility, and configurability in healthcare systems, according to the health, age, and disease conditions of the patient in various rehabilitation and nursing environments. The proposed knowledge-based physiological and homecare monitoring system integrates an efficient rule-based reasoning model and flexible knowledge rules for determining efficiently and rapidly the necessary physiological and medication treatment procedures based on software modules, video camera sensors, communication devices, and physiological sensor information. By integrating the proposed knowledge-based system with software components, video cameras, a set of physiological sensor modules, network communication devices, and ubiquitous handheld devices, an intelligent homecare system was implemented to provide the monitoring functionalities of patient activities and behaviors in various rooms and locations, as well as to monitor ubiquitously patient video records and patient physiological situations, treatment records, and warning conditions. The proposed knowledge-based system framework can offer significant flexibility for improving and extending the system further to meet new patient (such as chronic disease patients, older patients, and rehabilitation patients) and caregiver health care and monitoring demands by updating the knowledge rules in the inference mechanism. In addition, all of the functional components offer reusability, portability, and extensibility for homecare system developers to design and implement customized homecare systems to satisfy the various demands of different patients and caregivers from ubiquitous healthcare and monitoring aspects. The implementation and experimental results demonstrate that the proposed intelligent homecare system framework is efficient, feasible, extensible, and customizable for developing customized ubiquitous homecare systems to meet various demands of patients and caregivers under various rehabilitation and nursing conditions.

## Figures and Tables

**Table 1.** List of scenario cases provided by using the proposed homecare system framework.

**Scenario case**	**Actor**	**Brief semantics**
Record video of patient's activities	Patient	• The system is capable of recording the patient's rehabilitation in various home settings.
Record patient's physiological data & treatment records	Patient	• The system is capable of recording patient's physiological data from home sensors to assist the patient in understanding health and rehabilitation conditions.
Monitor & alert patient's treatment process	Patient & Caregiver	• The system establishes warning conditions for patients according to their physiological records and alerts them to follow rehabilitation treatment plans as determined by the knowledge-based system.
		• The system alerts caregivers when patients' health statuses deteriorate according to warning conditions determined by the knowledge-based system.
Retrieve & display video of patient's activities	Caregiver	• The system supports the caregiver's observation by retrieving the patient's videos for mobile terminal devices and Web browsers.
Retrieve patient's physiological data & treatment records	Caregiver	• The system transmits the patients' monitoring videos as well as health and rehabilitation conditions to the caregiver's remote browser and monitoring device.
Remote monitoring of patient's video records	Caregiver	• The system provides a monitoring function for caregivers to observe patients' video records of rehabilitation activities.
Remote monitoring patient's treatment process	Caregiver	• The system enables caregivers to monitor a patient's treatment process.
Remote monitoring of patient's physiological data & warning states	Caregiver	• The system provides a monitoring function for caregivers to monitor a patient's health and treatment conditions according to physiological data and treatment records.

**Table 2.** Experimental data of patients' abnormal physiological and treatment alarm situations of the proposed homecare system.

**Test cases**	**No. of occurred abnormal physiological conditions**	**No. of corrected determined abnormal physiological conditions**	**No. of occurred irregular treatment conditions**	**No. of corrected detected irregular treatment conditions**
Patient 1	3	3	4	4
Patient 2	7	7	2	2
Patient 3	7	7	7	7
Patient 4	5	5	8	8
Patient 5	3	3	2	2
Total events	25	25	23	23
Detection accuracy of alarm situations	100%			
